# Mitochondrial ROS potentiates indirect activation of the AIM2 inflammasome

**DOI:** 10.3389/fmicb.2014.00438

**Published:** 2014-08-20

**Authors:** Deborah D. Crane, Timothy J. Bauler, Tara D. Wehrly, Catharine M. Bosio

**Affiliations:** Immunity to Pulmonary Pathogens Section, Laboratory of Intracellular Parasites, Rocky Mountain Laboratories, National Institute of Allergy and Infectious Diseases – National Institutes of HealthHamilton, MT, USA

**Keywords:** inflammasome, tularemia, macrophage, reactive oxygen species

## Abstract

Activation of the inflammasome is important for the detection and clearance of cytosolic pathogens. In contrast to avirulent *Francisella novicida* (*Fn*), infection with virulent *Francisella tularensis* ssp *tularensis* does not trigger activation of the host AIM2 inflammasome. Here we show that differential activation of AIM2 following *Francisella* infection is due to sensitivity of each isolate to reactive oxygen species (ROS). ROS present at the outset of *Fn* infection contributes to activation of the AIM2 inflammasome, independent of NLRP3 and NADPH oxidase. Rather, mitochondrial ROS (mROS) is critical for *Fn* stimulation of the inflammasome. This study represents the first demonstration of the importance of mROS in the activation of the AIM2 inflammasome by bacteria. Our results also demonstrate that bacterial resistance to mROS is a mechanism of virulence for early evasion of detection by the host.

## INTRODUCTION

The innate immune system recognizes pathogen associated molecular patterns (PAMPs) using germline encoded pattern recognition receptors (PRRs) to initiate immune responses to pathogens. NOD-like receptors (NLR) and AIM2 are cytosolic PRRs that function as a scaffold to promote assembly of the inflammasome. The inflammasome is an innate immune signaling complex which activates caspase-1, resulting in cleavage of caspase-1 into two subunits, in response to intracellular pathogens and danger signals (Franchi et al., 2012). Activation of caspase-1 results in pyroptosis, a proinflammatory cell death process, and cleavage and secretion of the proinflammatory cytokines IL-1β and IL-18. Activation of the inflammasome is essential for clearance of many intracellular bacteria including *Shigella flexneri, Listeria monocytogenes, Legionella pneumophila, Salmonella typhimurium*, and avirulent *Francisella novicida* (*Fn*; Mariathasan et al., 2005). It is suspected that the ability of fully virulent cytosolic pathogens, e.g., virulent *F. tularensis* ssp. *tularensis*, to escape detection by the inflammasome is a critical component of virulence.

The sole known inflammasome activated following infection of mouse cells with *Fn* is the AIM2 inflammasome (Fernandes-Alnemri et al., 2010). Currently, the only known ligand for AIM2 is DNA (Fernandes-Alnemri et al., 2009). Thus, activation of AIM2 requires ready availability of bacterial DNA to the host cytosol. It has been suggested that fully virulent strains of *F. tularensis*, e.g., SchuS4, evade activating AIM2. Together this implies that there is a difference in the availability of *Fn* DNA as compared to SchuS4 DNA for detection by AIM2. However, there is no explanation for how *Fn* DNA becomes available to AIM2 or why SchuS4 DNA is not accessible for detection by the AIM2 inflammasome. In this study we provide clear evidence that activation of the inflammasome following *Fn* infection is due to heightened sensitivity of *Fn* to membrane damaging reactive oxygen species (ROS) as compared to SchuS4. We show that ROS generated by NADPH oxidase is not the source of ROS required for detection of Fn. Rather, mitochondrial derived ROS (mROS) is required for optimal activation of the inflammasome by Fn. This is the first example of mitochondria playing a role in the activation of the AIM2 inflammasome and explains the mechanism by which highly virulent bacteria successfully avoid triggering this important intracellular defense system.

## MATERIALS AND METHODS

### MICE AND GENERATION OF BONE MARROW DERIVED MACROPHAGES (BMM)

Specific pathogen free C57BL/6J mice were purchased from Jackson Laboratories (Bar Harbor, ME, USA). gp91/nos2^-/-^ were bred at Rocky Mountain Laboratories (RML). All research involving animals was conducted in accordance with Animal Care and Use guidelines under animal protocols approved by the Animal Care and Use Committee at RML. Bone marrow derived macrophages (BMM) were generated from femurs of mice as previously described (Crane et al., 2013).

### BACTERIA

Stock cultures of *F. tularensis* ssp. *tularensis* strain SchuS4 (Jeannine Peterson, CDC, Fort Collins, CO, USA) and *Fn* strain U112 (Denise Monack, Stanford University, Stanford, CA, USA) were generated and utilized as previously described (Dreisbach et al., 2000; Svensson et al., 2012). Briefly, bacteria were grown for 16 h in modified Mueller Hinton (MMH) broth. Then bacteria were aliquoted into 1 ml samples and frozen at -80^∘^C. Immediately prior to use, bacteria were rapidly thawed and diluted to the indicated MOI. Inoculum titers for each experiment were confirmed by plating the inoculum onto MMH agar, incubating plates at 37^∘^C and counting individual colonies. Titer of stock cultures varied less than 5% over a 12 month period. All experiments were performed under approved BSL-2 or BSL-3 safety protocols at RML.

### SENSITIVITY TO CHEMICALS

Sodium deoxycholate, SDS, and H_2_O_2_ (all from Sigma, St. Louis, MO, USA) and EDTA (Ambion, Grand Island, NY, USA) were diluted to the indicated concentration in PBS. Bacteria were added to each solution at a final concentration of 10^6^ bacteria/ml. Bacteria were incubated at 37^∘^C for 2 h with constant shaking. Then bacteria were serially diluted, plated on MMH agar, incubated at 37^∘^C and colonies were enumerated 48 h later.

### INFECTION OF BMM

Bone marrow derived macrophages were infected with the indicated multiplicity of infection (MOI) of *Fn* or SchuS4 as previously described (Griffin et al., 2013). Briefly, bacteria were diluted to the indicated MOI and added to BMM. BMM were incubated for 90 min at 37^∘^C/5% CO_2_. Then, bacteria containing medium was pipetted off and BMM were incubated with gentamicin (50 μg/ml) for 45 min. BMM were washed extensively with PBS and incubated in DMEM supplemented with 10% heat inactivated fetal bovine serum, L-glutamine, non-essential amino acids, and HEPES (cDMEM; all from Life Technologies). Intracellular bacteria were enumerated by lysing BMM with water and plating lysates on MMH agar as previously described (Bauler et al., 2011). Where indicated cells were pretreated with 3 mM *N*-aceytlcysteine (NAC; Sigma), 1 mM L-NMMA (Cayman Chemicals, Ann Arbor, MI, USA) or 250 μM mitoTempo (Enzo Life Sciences, Farmingdale, NY, USA) 16, 24, or 1 h prior to infection, respectively. Effective concentrations of NAC and L-NMMA used in this study were determined by their ability to inhibit production of ROS and RNS in BMM treated with LPS + IFN-γ (Crane, unpublished data). Various concentrations of mitoTEMPO were tested in BMM. Only concentrations of 250 μM and above had any effect on ROS production in cells (Bauler, unpublished data).

### WESTERN BLOTTING

Bone marrow derived macrophages lysates were generated using NuPage LDS Sample Buffer 24 h after infection (Life Technologies). Lysates were resolved on 4–12% NuPAGE gradient gels and proteins were transferred to PVDF membranes (Life Technologies). Membranes were blocked with 5%BSA in Tris Buffered Saline + 0.05% Tween 20 (TBST) and then were probed with antibodies to caspase-1 p10 or p20 (M-20, Santa Cruz Biotechnology, Dallas, TX, USA; MBL International, Woburn, MA, USA, respectively) and β-actin (13E5, Cell Signal Technologies) as previously described (Jones et al., 2010; Bauler et al., 2011).

### ANALYSIS OF IL-1β

IL-1β present in cell culture supernatants was assessed by ELISA 24 h after infection using commercially available kits and following manufacturer’s instructions (R&D Systems, Minneapolis, MN, USA).

### STATISTICAL ANALYSIS

Values are expressed as the mean of triplicate samples, unless otherwise noted. Statistical differences between two groups were determined using a two-tailed Student’s *t*-test. For comparison between three or more groups, analysis was done by one-way ANOVA followed by Tukey’s multiple comparisons test.

## RESULTS

### VIRULENT *F. tularensis* DOES NOT ACTIVATE THE INFLAMMASOME

**FIGURE 1:**
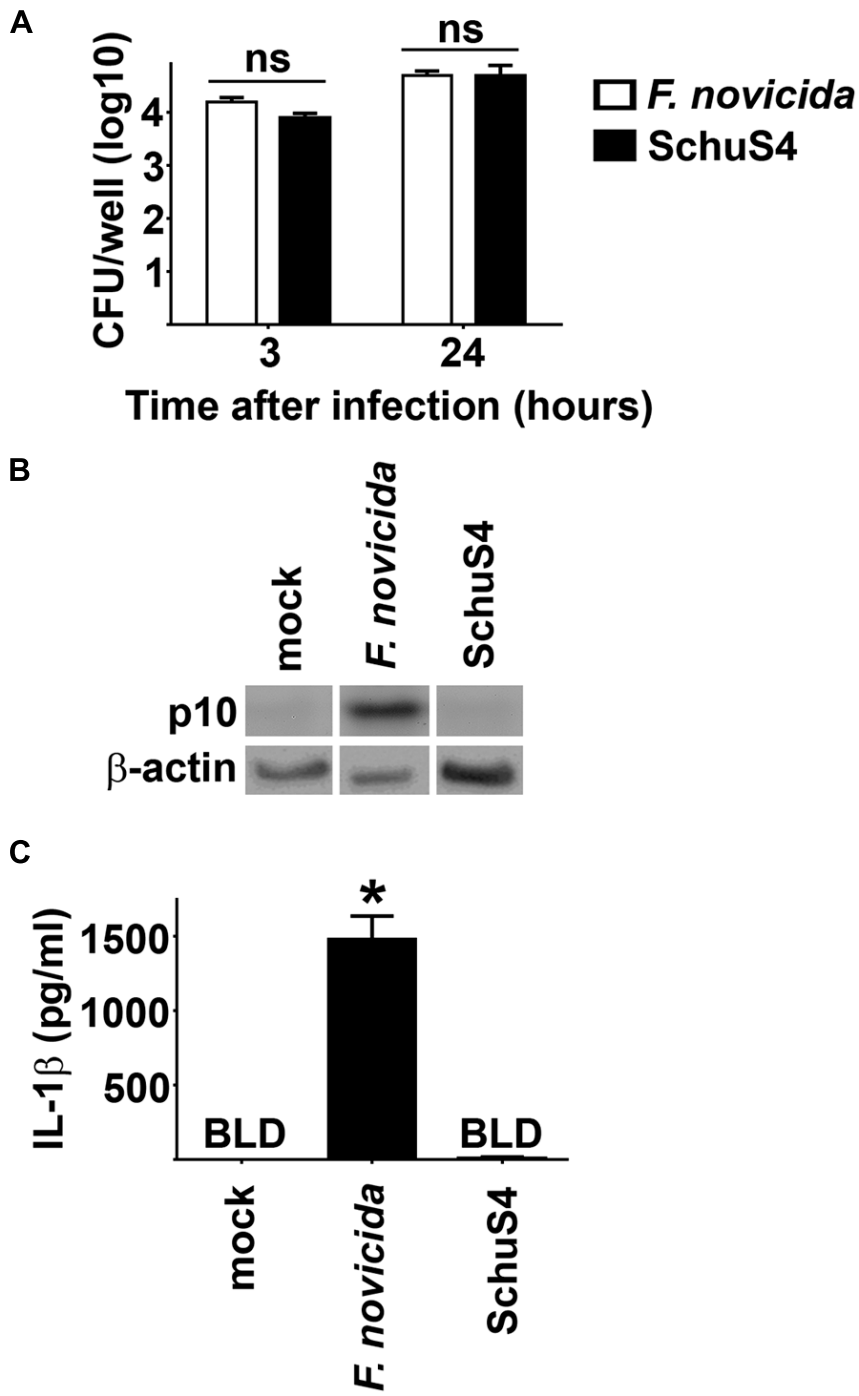
**Evasion of the inflammasome by virulent *Francisella* in bone marrow macrophages (BMM).** BMM were infected with *Fn* MOI 250 and SchuS4 MOI 150. MOI were selected to match uptake of *Fn* and SchuS4. **(A)** Intracellular bacteria were enumerated at the indicated times after infection. **(B)** Western blots of cell lysates were probed for caspase-1 p10 and actin. **(C)** Culture supernatants were assessed for IL-1β by ELISA. Error bars represent SD. ns, not significant, BLD, below level of detection, *p < 0.05 compared to all other groups. Data are representative of two independent experiments.

Inflammasomes are important components of innate immunity that promote secretion of proinflammatory cytokines such as IL-1β in response to intracellular infection. One mechanism of virulence of SchuS4 is its ability to escape detection by the host cell. However, the ability of SchuS4 to evade triggering the host inflammasome has not been described. Thus, we first determined if SchuS4 activated the inflammasome in BMM and compared this response to cells infected with Fn. MOIs of *Fn* and SchuS4 were adjusted to result in similar uptake (**Figure 1A**). *Fn* and SchuS4 replicated to similar numbers BMM (**Figure 1A**). In agreement with previously published data, infection with *Fn* resulted in cleavage of caspase-1 as noted by the appearance of caspase-1 p10 subunit. Activation of Caspase-1 was further confirmed by secretion of IL-1β among *Fn* infected cells (**Figures 1B,C**). In contrast, SchuS4 infection did not induce cleavage of caspase-1 or secretion of IL-1β (**Figures 1B,C**). We also examined inflammasome activation by SchuS4 at multiple time points after infection, i.e., 8, 16, 24, and 48 h and at various MOIs from 10 to 1000. At no time or MOI did we detect cleavage of Caspase-1 or secretion of IL-1β from SchuS4 infected cells (Crane et al., unpublished data). Together this demonstrated that virulent SchuS4 does not activate the inflammasome in resting BMM.

### SchuS4 AND *Fn* EXHIBIT DIFFERENTIAL SENSITIVITY TO MEMBRANE DAMAGING CHEMICALS

**FIGURE 2:**
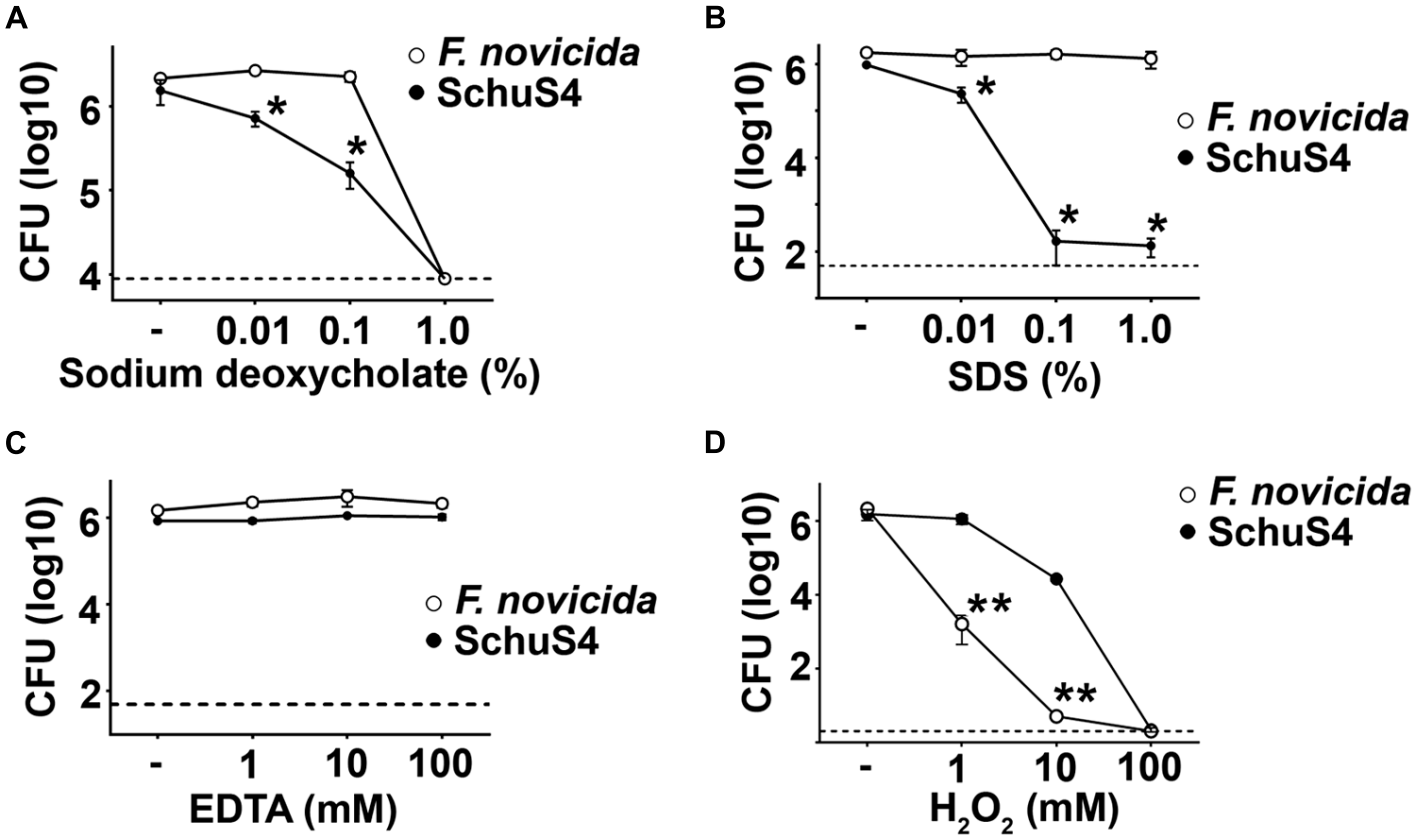
***Francisella novicida* exhibits greater sensitivity to H_**2**_O_**2**_ than SchuS4.** Bacteria (10^6^/ml) were added to solutions of **(A)** sodium deoxycholate, **(B)** SDS, **(C)** EDTA, or **(D)** H_2_O_2_ incubated at 37^∘^C for 2 h. Then, bacteria were serially diluted and plated on MMH agar to enumerate colonies. Dashed lines represent the limit of detection. Error bars represent SD. *p < 0.05 compared to *Fn*. **p < 0.05 compared to SchuS4. Data are representative of two experiments of similar design.

Activation of the AIM2 inflammasome is dependent upon release of bacterial DNA into the host cytosol. Therefore, there must be significant perturbations of the bacterial membrane that allow release of their DNA into the host cytosol. In support of this hypothesis, it has been shown that defined mutants of *Fn* and LVS which trigger activation of the inflammasome above that observed following infection with wild type (WT) parental strains rapidly lyse in the cytosol (Peng et al., 2011). This increased lysis was suggested to be a result of decreased stability of the bacterial cell membranes. Considering the correlation of poor membrane stability and activation of the inflammasome by *Fn* and LVS, we hypothesized that the differential activation of the inflammasome by *Fn* and SchuS4 in our study may be a result of reduced stability and/or increased sensitivity of *Fn* membranes to membrane perturbing compounds. To test this hypothesis, we exposed *Fn* and SchuS4 to various detergents and chemicals that are known to disturb bacterial membrane integrity. SchuS4 exhibited greater sensitivity to detergents sodium deoxycholate and SDS (**Figures 2A,B**). Neither SchuS4 nor *Fn* exhibited significant sensitivity to calcium and magnesium chelator EDTA (**Figure 2C**). In addition to detergents and other lysis inducing agents, ROS are known to destabilize bacterial membranes (Fang, 2004). Further, ROS are an important part of host defense and are produced by host cells via a variety of mechanisms to control microbes. The relative sensitivity of *Fn* to H_2_O_2_ compared to virulent SchuS4 is not known. Thus, we compared the ability of H_2_O_2_ to kill *Fn* versus SchuS4. SchuS4 was significantly more resistant to H_2_O_2_ than *Fn* (**Figure 2D**). Since *Fn* was more resistant to sodium deoxycholate and SDS compared to SchuS4, the heightened sensitivity of *Fn* to the ROS H_2_O_2_ was not due to a general defect in membrane integrity of this bacterium.

### INHIBITION OF ROS HINDERS *Fn* DRIVEN INFLAMMASOME ACTIVATION

**FIGURE 3:**
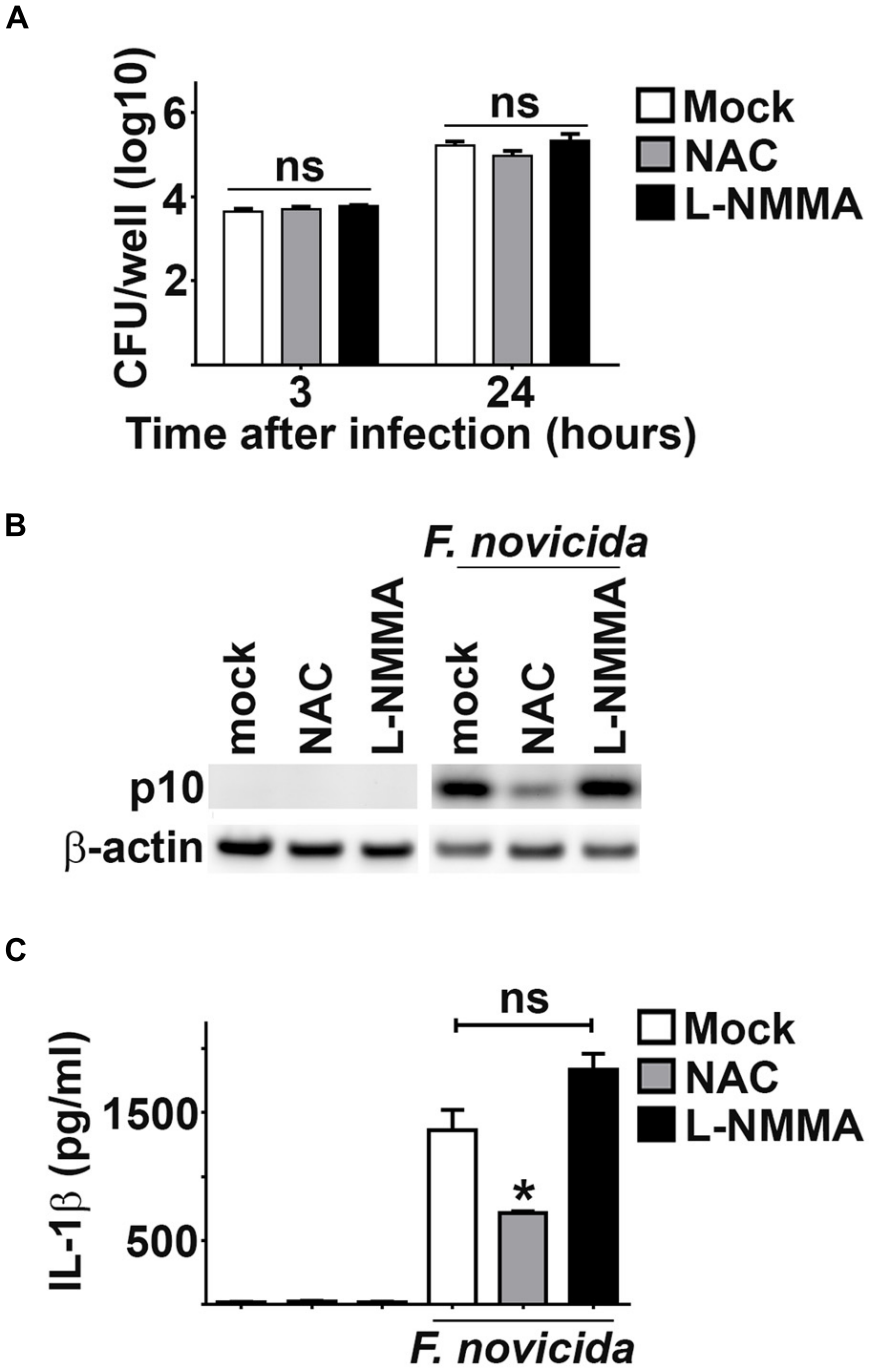
**Inhibition of ROS suppresses *Fn* driven inflammasome activation.** As indicated, BMM were treated with NAC (3 mM), L-NMMA (1 mM) or water (vehicle control; mock) 16 or 24 h prior to infection with *Fn* MOI 250, respectively. **(A)** Intracellular bacteria were enumerated at the indicated times after infection. **(B)** Western blots of cell lysates were probed for caspase-1 p10 and actin. **(C)** Culture supernatants were assessed for IL-1β by ELISA. Error bars depict SD. ns, no significant difference between mock and L-NMMA treated cells. *p < 0.05 compared to mock and L-NMMA treated cells. Data are representative of three experiments of similar design.

Given the increased sensitivity of *Fn* to ROS compared to SchuS4, we postulated that interaction of ROS with *Fn* may contribute to the presence of AIM2 activating DNA. To test this idea we inhibited ROS among *Fn* infected cells and assessed inflammasome activation via cleavage of caspase-1 and secretion of IL-1β into culture supernatants. Treatment of BMM with the ROS inhibitor NAC did not significantly affect uptake or replication of *Fn* (**Figure 3A**). Inhibition of ROS markedly reduced cleavage of caspase-1 and significantly reduced the amount of IL-1β secreted by *Fn* infected cells (**Figures 3B,C**). Reactive nitrogen species (RNS) have also been implicated in disrupting bacterial cell membranes which could allow release of bacterial DNA. Therefore, we also inhibited RNS in *Fn* infected cells and measured inflammasome activation. Similar to cells treated with NAC, the RNS inhibitor L-NMMA had no effect on uptake or replication of *Fn* in BMM (**Figure 3A**). Interference with RNS did not alter the presence of caspase-1 p10 nor secretion of IL-1β among *Fn* infected cells (**Figures 3B,C**). Thus, sensitivity to ROS, but not RNS, contributes to the ability of *Fn* to drive activation of the inflammasome.

### ABSENCE OF NADPH OXIDASE DOES NOT AFFECT *Fn* INFLAMMASOME ACTIVATION

**FIGURE 4:**
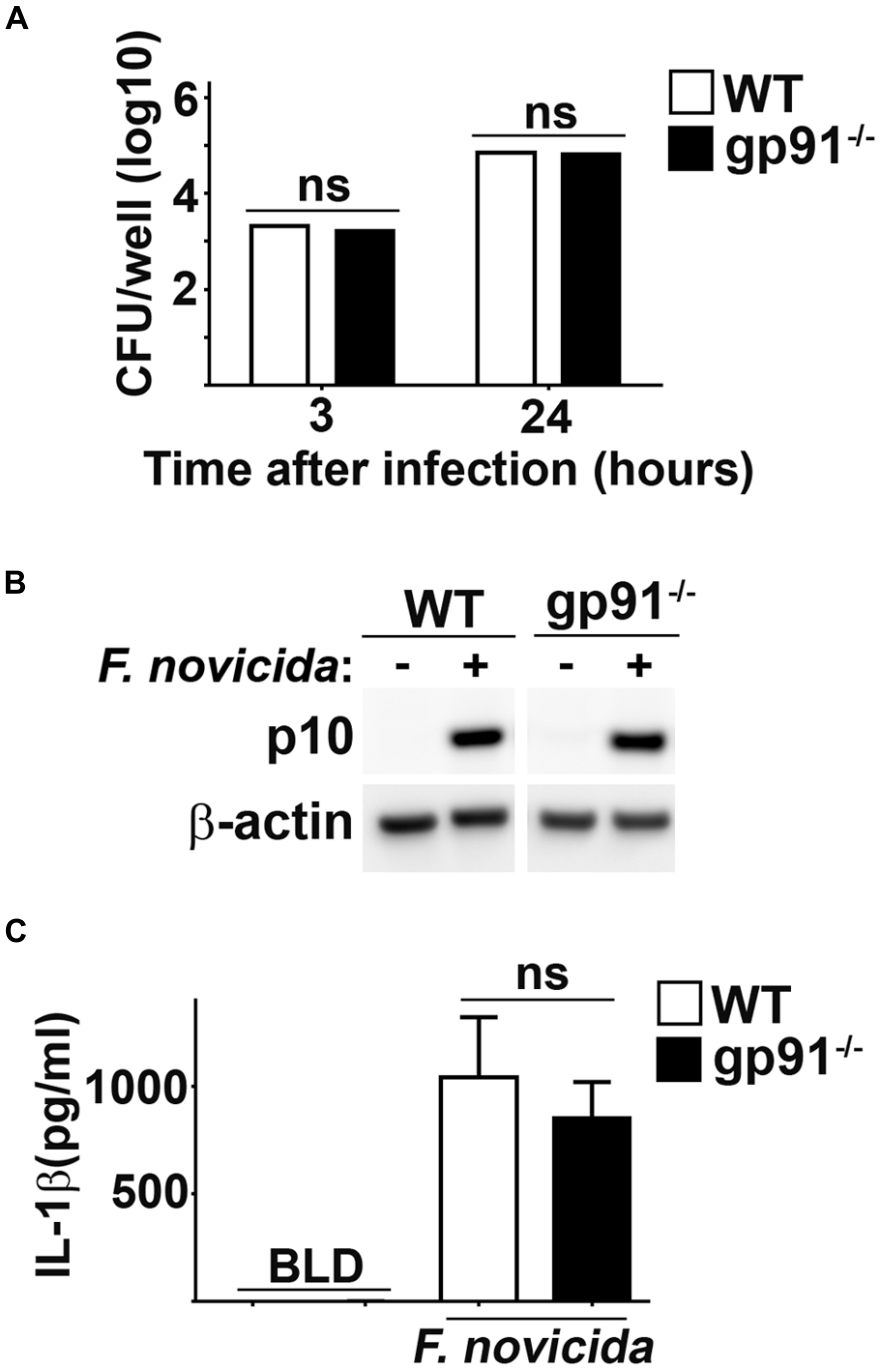
**NADPH oxidase does not contribute to *Fn* mediated activation of the inflammasome.** BMM from wild type (WT) or gp91/nos2^-/-^ (gp91^-/-^) mice were infected with *Fn* MOI 250. **(A)** Intracellular bacteria were enumerated at the indicated times after infection. **(B)** Western blots of cell lysates were probed for caspase-1 p10 and actin. **(C)** Culture supernatants were assessed for IL-1β by ELISA. Error bars depict SD. ns, not significant. Data are representative of three experiments of similar design.

One of the most common sources of ROS in host cells is that derived from activity of the NADPH oxidase system. *Fn* has been shown to inhibit NADPH assembly and function, arguing against a role for this host complex in generating ROS that contributes to *Fn* mediated inflammasome activation (Mohapatra et al., 2010). However, it is possible that this process may be inefficient allowing for some ROS producing NADPH complexes to form which could damage bacterial membranes, resulting in inflammasome activation. To determine if ROS derived from NADPH oxidase contributed to *Fn* driven inflammasome activation, we infected macrophages deficient for the gp91 subunit of NADPH oxidase (gp91^-/-^) which cannot assemble functional NADPH oxidase complexes. WT and gp91^-/-^ BMM phagocytosed and supported replication of *Fn* similarly (**Figure 4A**). Absence of gp91 did not impact the ability of *Fn* to provoke cleavage of caspase-1 or secretion of IL-1β (**Figures 4B,C**). Therefore, NADPH oxidase derived ROS does not contribute to *Fn* mediated inflammasome activation.

### mROS IS REQUIRED FOR ACTIVATION OF INFLAMMASOME BY *Fn*

**FIGURE 5:**
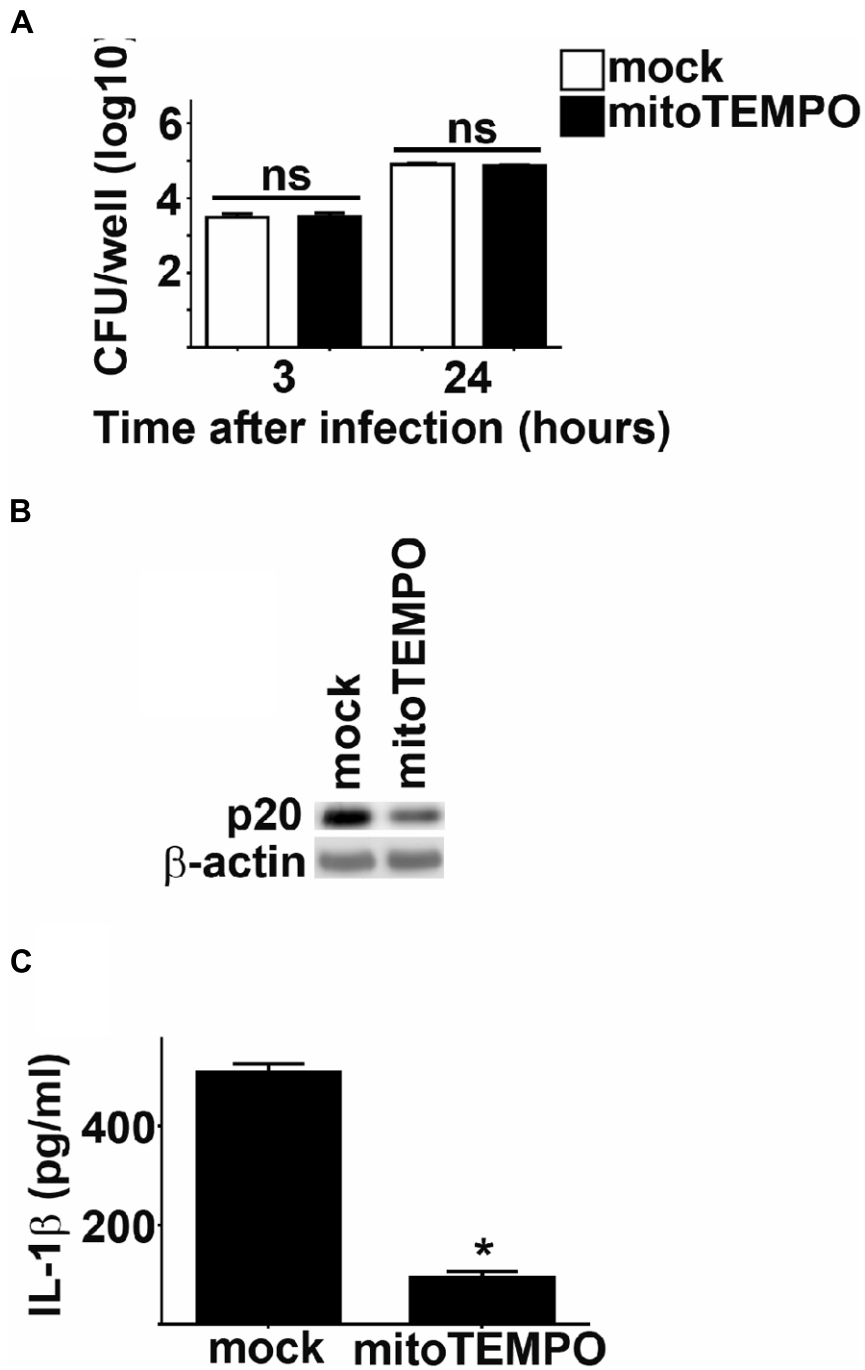
**mROS potentiates *Fn* mediated inflammasome activation.** BMM were treated with mitoTEMPO or DMSO (vehicle control; mock) 1 h prior to infection with *Fn* MOI 250. **(A)** Intracellular bacteria were enumerated at the indicated times after infection. ns, not significant. **(B)** Western blots of cell lysates were probed for caspase-1 p20 and actin. **(C)** Culture supernatants were assessed for IL-1 by ELISA. Error bars depict SD. ns, no significant difference between mock treated cells. *p < 0.05 compared all mock treated cells. Data are representative of two experiments of similar design.

In addition to the NADPH oxidase complex, mitochondria serve as an important source of ROS in host cells (West et al., 2011b). Since NADPH oxidase derived ROS did not play a role in *Fn* mediated inflammasome activation, we hypothesized that mROS may contribute to this process. We tested this hypothesis by comparing activation of the inflammasome in *Fn* infected cells treated with the mitochondrial specific ROS scavenger mitoTEMPO to mock treated controls (Dikalova et al., 2010). Treatment with mitoTEMPO had no effect on the uptake or replication of *Fn* in BMM (**Figure 5A**). However, inhibition of mROS reduced cleavage of caspase-1 and significantly reduced secretion of IL-1β by *Fn* infected cells (**Figures 5B,C**). mROS has also been implicated in activation of the NLRP3 inflammsome (Heid et al., 2013). Others have routinely demonstrated that activation of the inflammasome by *Fn* in mouse cells is mediated exclusively by AIM2 and not NLRP3 (Fernandes-Alnemri et al., 2010; Atianand et al., 2011). Nevertheless, given the association of mROS with the NLRP3 inflammasome, we also assessed if NLRP3 contributed to cleavage of caspase-1 and secretion of IL-1β among *Fn* infected cells. *Fn* infection resulted in similar activation cleavage of caspase-1 and secretion of IL-1β in NLRP3 deficient BMM compared to WT BMM. Thus, in agreement with previously published work, NLRP3 did not contribute to inflammasome activation among *Fn* infected cells (Bauler and Bosio, unpublished data). This confirmed that the inhibition of mROS in *Fn* infected cells was not be due to interference with NLRP3 driven inflammasome activation. Thus, mROS is required for optimal activation of the AIM2 inflammasome following *Fn* infection.

## DISCUSSION

Data presented herein demonstrate that mROS indirectly contributes to the activation of the AIM2 inflammasome following *Fn* infection. We proposed that the mechanism by which ROS contributes to AIM2 inflammasome activation was by destabilizing bacterial membranes allowing release of the AIM2 ligand, bacterial DNA, into the cytosol. This hypothesis was first supported by our observation that *Fn* was significantly more sensitive to the ROS H_2_O_2_ compared to SchuS4. Differential sensitivity to H_2_O_2_ among virulent SchuS4, *F. tularensi*s ssp. *holarctica* strain FSC200, and attenuated live vaccine strain (LVS) has been previously reported (Lindgren et al., 2007, 2011). Increased sensitivity of FSC200 and LVS to H_2_O_2_ compared to SchuS4 was not correlated with differential expression of genes known to neutralize ROC, e.g., katG and super oxide dismutase (SOD). Rather, sensitivity of FSC200 and LVS to H_2_O_2_ was attributed to their ability to take up and store higher amounts of iron, since iron uptake enhances toxicity of H_2_O_2_ (Lindgren et al., 2011). The specific genes mediating differences in iron uptake among FSC200, LVS, and SchuS4 that contribute to H_2_O_2_ toxicity have not been identified, nor has direct comparison of iron acquisition among *Fn* and SchuS4 been reported. Thus, it is possible that the increased sensitivity of *Fn* to H_2_O_2_ observed herein could be attributed to the ability of the bacterium to collect iron more efficiently than SchuS4. *Fn* possess similar operons and genes required for iron acquisition as SchuS4 (Ramakrishnan et al., 2012). Thus, identification of the potential role for iron uptake or other regulators of sensitivity to H_2_O_2_ will likely require screening libraries of defined *Fn* and SchuS4 mutants.

In addition to a role for iron in sensitivity to H_2_O_2_, difference in the expression of antioxidants among *Fn* and SchuS4 may explain their varied resistance to H_2_O_2_. *Fn* and SchuS4 carry genes that encode an array of antioxidants capable of neutralizing ROS and there are no apparent differences in the presence or absence of these genes between *Fn* or SchuS4. However, it is possible that SchuS4 may express higher concentrations of one or more of these proteins compared to *Fn* at the outset of infection. ROS can also affect bacterial viability via oxidation of bacterial lipids and/or misfolding of membrane proteins resulting in perturbation of bacteria membranes (Nathan and Cunningham-Bussel, 2013). We have shown that lipids isolated from *Fn* differ in their ability to provoke cytokine responses in macrophages compared to lipids isolated from SchuS4 (Crane et al., 2013). This suggested that lipid species present in *Fn* are different than those found in SchuS4. Thus, it is possible that lipids or lipoproteins found in *Fn* membranes are more susceptible to oxidation by ROS than those found in SchuS4. Alternatively, there may be variation in key membrane associated proteins among *Fn* as compared to SchuS4 that are misfolded in the presence ROS resulting in disruption of membrane integrity.

Regardless of the specific bacterial features that contribute to the increased sensitivity of *Fn* to ROS compared to SchuS4, we demonstrate that ROS plays an integral role in *Fn* mediate activation of the inflammasome, as measured by cleavage of Capsase-1. We also established that the source of ROS in host cells that contributed to activation of the AIM2 inflammasome was mROS. Initially, a role for mROS in *Fn* driven inflammasome activation was unexpected. However, it has been suggested that mROS is important for combatting infection mediated by intracellular pathogens (Arsenijevic et al., 2000). Further, mROS can be activated following ligation of TLR2 or TLR4 (West et al., 2011a). *Fn* mediated inflammatory responses are largely dependent on TLR2, whereas SchuS4 fails to trigger pro-inflammatory responses (Bosio et al., 2007; Bauler et al., 2011). Therefore, we hypothesize that the inflammatory nature of Fn, likely triggering through TLR2 or other PRRs, contributes to the presence of mROS. Alternatively, low levels of mROS may be present in the host cell prior to infection. Regardless of the means by which mROS is made available, we propose that mROS acts to destabilize *Fn* membranes allowing release of bacterial DNA into the cytosol subsequently resulting in AIM2 inflammasome activation.

Together these data describe an unexpected and important difference in the ability of avirulent *Fn* and virulent SchuS4 to trigger inflammasome activation. Furthermore, our data also suggest that the inability of SchuS4 to trigger inflammasome activation is directly associated with its enhanced resistance to ROS compared to Fn. Identification of the deficiencies in *Fn* resulting in its susceptibility to ROS and/or the molecules present in SchuS4 that augment its resistance to ROS may reveal novel targets for effective therapeutics against tularemia or other intracellular pathogens.

## Conflict of Interest Statement

The authors declare that the research was conducted in the absence of any commercial or financial relationships that could be construed as a potential conflict of interest.
